# Involvement of long non-coding RNA (lncRNA) MALAT1 in shear stress regulated adipocyte differentiation

**DOI:** 10.3389/fbioe.2025.1570518

**Published:** 2025-05-06

**Authors:** Justin Caron, Marjan Ghanbariabdolmaleki, Madison Marino, Chong Qiu, Bo Wang, Michael Mak, Shue Wang

**Affiliations:** ^1^ Department of Chemistry, Chemical and Biomedical Engineering, University of New Haven, West Haven, CT, United States; ^2^ Department of Forensic Science, University of New Haven, West Haven, CT, United States; ^3^ Joint Department of Biomedical Engineering, Marquette University and the Medical College of Wisconsin, Milwaukee, WI, United States; ^4^ Department of Pharmacological Sciences, Renaissance School of Medicine, Stony Brook University, Stony Brook, NY, United States

**Keywords:** shear stress, adipocyte differentiation, osteogenic differentiation, long non-coding RNA, lncRNA MALAT1, nanobiosensor

## Abstract

Adipocyte differentiation plays an important role in bone remodeling due to secretory factors that can directly modulate osteoblast and osteoclast, thus affecting overall bone mass and skeletal integrity. Excessive adipocyte differentiation within the bone marrow microenvironment can lead to decreased bone mass, eventually causing osteoporosis. The mechanical microenvironment of bone marrow, including fluid shear, maintains the balance of adipocyte and osteoblast differentiation during bone remodeling. However, how mechanical cues interact with long noncoding RNA (lncRNA) and regulate adipocyte differentiation remains unexplored. In this study, we investigated the mechanosensitive role of lncRNA MALAT1 during mesenchymal stem cells (MSCs) adipocyte differentiation. By applying physiologically relevant shear stress, MSCs experienced morphological changes and adipocyte differentiation differences. Shear stress inhibits adipocyte differentiation of MSCs, demonstrated by reduced oil-red-o-stained lipid droplets. Silencing MALAT1 also results in reduced adipocyte differentiation. By leveraging a novel gapmer double stranded locked nuclei acid nanobiosensor, we showed that shear stress inhibits MALAT1 expression, with significantly reduced fluorescence intensity. Our findings indicate that shear stress influences adipocyte differentiation mainly through the downregulation of MALAT1, highlighting a significant interplay between biophysical cues and lncRNAs. This interaction is crucial for understanding the complexities of bone remodeling and the potential therapeutic targeting of lncRNAs to treat bone-related disorders.

## Introduction

Mesenchymal stem cells (MSCs) are multipotent, self-renewing stem cells that are able to differentiate into numerous cell types, including osteoblasts, adipocytes, chondrocytes, hepatocytes, cardiomyocytes, and nerve cells with target specific biochemical factors (Pittenger et al., 2019; Wang and Chen, 2013). Increased evidence has shown that MSCs have been the favorite cell sources for the application of tissue engineering and regenerative medicine due to their remarkable properties (Fitzsimmons et al., 2018). Various studies have shown that MSCs have been applied in repairing bone (Lin et al., 2019; Zhang et al., 2018), cartilage (Fahy et al., 2018), adipose (Li and Wu, 2020), nerve (Fernandes et al., 2018), and other organs (Han et al., 2019; Leach and Whitehead, 2017). However, the success of MSCs-based cell therapy and tissue engineering is highly dependent on MSCs fate determination or commitment. Thus, how to precisely control MSCs differentiation into desired lineage or phenotype is critical for MSCs-based therapy. Over the last few decades, unremitting efforts have been devoted to understanding biochemical signals that regulate MSCs commitment. A number of critical signaling pathways are identified and involved in regulating MSCs lineage commitment, including bone morphogenetic protein (BMP) signaling, Wnt signaling, and Notch signaling (Guilak et al., 2009; Wang et al., 2012; Heo et al., 2016). Recently, accumulating data suggested that long non-coding RNAs (lncRNA) emerge as novel regulators of numerous biological processes, including embryonic development (Yan et al., 2013), cancer progression (Li et al., 2016), and stem cell fate determination (Aich and Chakraborty, 2020). LncRNAs are transcripts of longer than 200 nucleotides that are not translated into proteins but play important regulatory roles in transcriptional and post-transcriptional regulation. Recent studies have shown that several lncRNAs are involved in the regulation of stem cell fate commitment (Lanzillotti et al., 2021). For example, it is reported that lncRNA-LULC active smooth muscle differentiation of adipose-derived MSCs by upregulation of BMP9 expression; lncRNA MALAT1 (Yi et al., 2019), MSC-AS1 (Zhang N. et al., 2019), lncRNA-OG, H19 (Zhou et al., 2021), NEAT1 (Zhang Y. et al., 2019) promote osteogenic differentiation either through miRNA-related regulation or chromatin remodeling (Li et al., 2020). lncRNA HOTAIR (Potolitsyna et al., 2022; Li et al., 2021), GAS5 (Li et al., 2018), and ANCR (Ju et al., 2019) were reported to negative regulate adipocyte differentiation. Over the past decade, extensive studies have performed to investigate how mechanical cues like shear, stiffness, and topography affect MSCs fate determination (Xue et al., 2016; Xue et al., 2017). Fluid shear stress (SS), resulting from exposure to vascular or interstitial fluid flow, is a key biophysical factor that influences mesenchymal stem cell (MSC) fate. However, it has been studied less compared to other mechanical cues, despite its significant impact on MSC differentiation and behavior. Our recent study showed physiological relevant shear stress enhance MSCs osteogenic differentiation and partially through Notch activation (Zhao et al., 2022a). However, the effects of shear stress on adipocyte differentiation are obscure and have controversial results. In a study by Adeniran-Catlett et al., the authors demonstrated that shear stress (15 dyne/cm^2^, 1.5 Pa) enhance adipogenesis with increased accumulation of lipids (Adeniran-Catlett et al., 2016). However, in several other studies, it has been reported shear stress in the range of (6–10 dyne/cm^2^) suppressed MSCs adipogenesis as indicated by reduced lipid droplet accumulation (Choi et al., 2017). Interestingly, significantly lower shear stress (0.28 dyne/cm^2^) demonstrated a markable increase in lipid accumulation (Wu et al., 2011). Another study showed in obese patients, the wall shear stress of the vein is lower (lower than 0.25 Pa) compared to control group (0.3–0.6 Pa) (Wiewiora et al., 2013). Thus, it is intriguing to study the effect of shear at the range of 0.3–0.6 Pa on adipogenesis of MSCs, which mimicking the normal physiological relevant shear. Inspired by native physiological microenvironment, recent studies suggest that shear stress can act in synergism with other biochemical cues to augment the differentiation efficiency of MSCs. This combination effectively replicates *in vitro* conditions of MSCs. Here, we investigated shear stress in combination of biochemical factor, lncRNA MALAT1 in regulating MSCs adipocyte differentiation. We first investigated and compared the effects of fluid shear stress on MSCs proliferation and adipocyte differentiation. The phenotypic behaviors, including morphology, proliferation, and differentiation were characterized and compared. We further studied the role of lncRNA MALAT1 in regulating adipocyte differentiation. Finally, we examined the mechanosensitive role of MALAT1 using a nanobiosensor. Our experimental results provide convincing evidence supporting that physiologically relevant shear stress inhibits adipocyte differentiation of MSCs with significantly reduced accumulation of lipid droplets. Silencing MALATs results in reduced adipocyte differentiation. Our study suggests that shear stress inhibits MSCs adipocyte differentiation through the downregulation of lncRNA MALAT1. This study will add new information on mechanosensitive role of MALAT1 in regulating MSCs lineage determination.

## Materials and methods

### Cell culture and reagents

**FIGURE 1:**
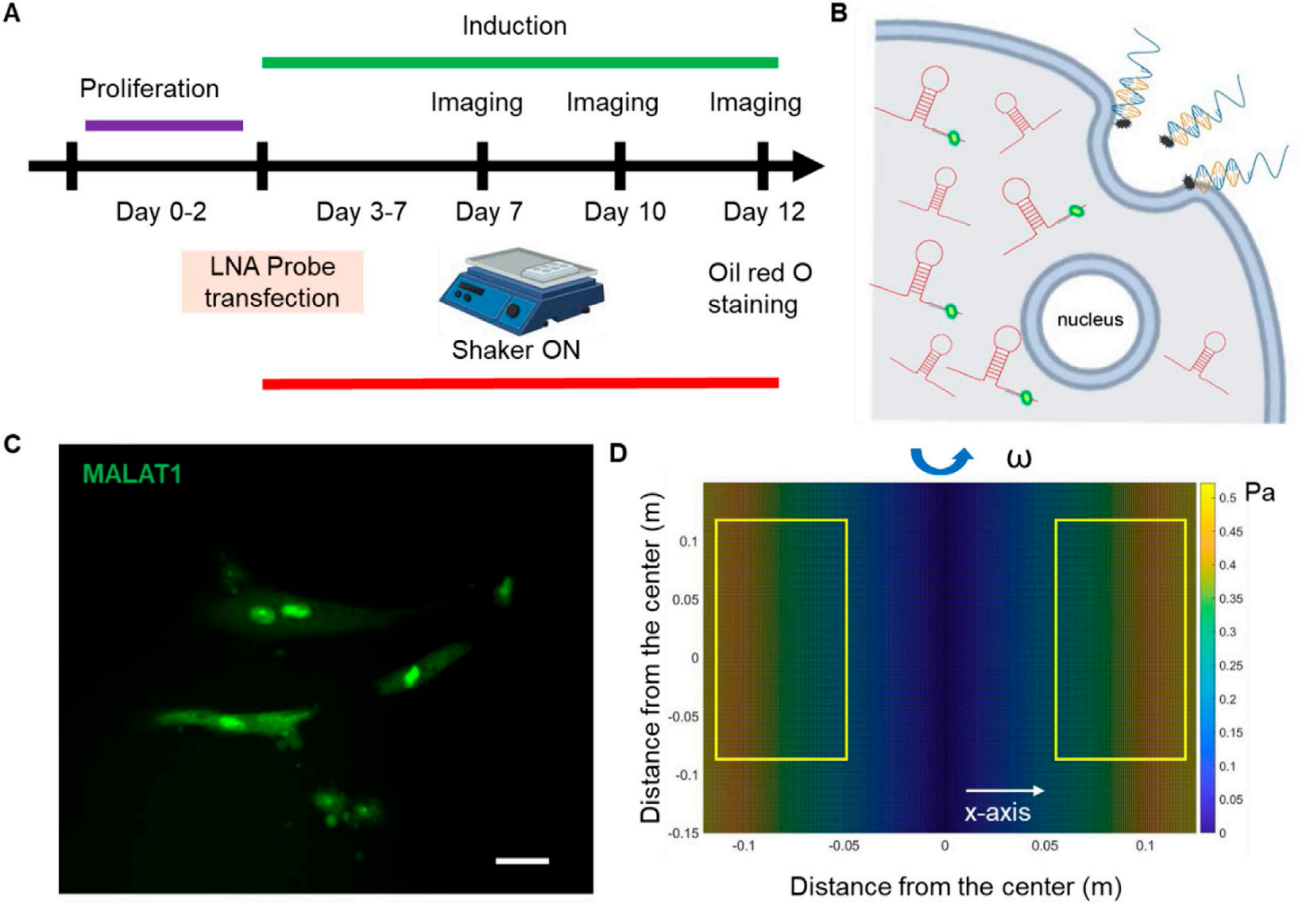
Overview of experimental design. **(A)** Experimental design. **(B)** Schematic illustration of endocytic uptake of LNA nanobiosensor by MSCs for lncRNA detection. Once internalized, the LNA detecting probe is displaced from the dsLNA complex and binds to the target lncRNA, allowing the fluorophore to fluorescence. **(C)** Representative fluorescence image of lncRNA MALAT1 expression in MSCs. Scale bar: 50 µm. **(D)** Simulated distribution of orbital shear stress. Yellow labeled rectangles indicate the location of well-plates. The estimated shear stress was in the range of 0.3–0.6 Pa.

Human bone marrow-derived MSCs were acquired from Lonza, which were originally isolated from normal adult human bone marrow withdrawn from bilateral punctures of the posterior iliac crests of volunteers. MSCs were cultured using basal medium MSCBM (PT-3238, Lonza) with GA-1000, L-glutamine, and mesenchymal cell growth factors (PT-4105, Lonza). Cells were cultured in tissue culture dishes in a humidified incubator at 37°C with 5% CO_2_ with medium change every 3 days. Cells were passaged using 0.25% EDTA-Trypsin (Invitrogen) and passage 2-7 were used for the experiments. Adipogenic induction medium were purchased from Lonza (PT-4135). Induction medium was prepared by adding Adipogenic Differentiation SignleQuots Supplements (PT-4135) which include h-insulin, L-glutamine, MCGS, dexamethasone, indomethacin, IBMX, and GA-1000, into 170 mL of adipogenic differentiation medium (PT-3102B). For adipocyte differentiation, MSCs were seeded at a concentration of 400 cells/mm^2^ with a volume of 500 μL basal medium in 24 well-plates. Cells were maintained in basal medium until they reached 70%–80% confluency. Then the basal medium was replaced with adipocyte differentiation medium and refreshed every 3 days. The adipocyte differentiation was evaluated after 5, 7, and 10 days of induction. Figure 1A showed the experimental design process.

### Design process of LNA/DNA nanobiosensor

This dsLNA nanobiosensor is a complex of a detection LNA probe and a quencher probe, with the length of 30- and 15- oligonucleotide sequence, respectively. The detection probe is a single-stranded LNA/DNA, with LNA monomers at the two ends of the oligonucleotide sequence that was designed to be complementary of partial MALAT1 sequence, Supplementary Table S1. The sequence was selected and validated for its specificity using RNA fold and BLAST (Fasciano et al., 2023). The quencher probe is a 15-base pair single-stranded LNA/DNA, with a quenching dye at the 3′ end of the sequence. The detection probe will bind to the quencher probe to form stable dsLNA complex, initiating the quenching. Once the LNA complex is transfected into the cells, in the presence of the lncRNA target sequence, the LNA detection probe is thermodynamically displaced from the quencher, allowing the fluorophore to fluorescence, thus detecting lncRNA expression at the single cell level. This displacement is due to a larger difference in free binding energy between LNA detection probe to target lncRNA versus LNA probe to quencher.

### Preparation of double-stranded LNA probe

To prepare the LNA/DNA nanobiosensor, the LNA detecting probe and quencher probe were initially prepared in ×1 Tris EDTA buffer (pH = 8.0) at a concentration of 100 nM. The LNA probe and quencher were mixed at the ratio of 1:2 and incubated at 95°C in a dry water bath for 5 min and cooled down to room temperature over the course of 2 h. Once cooled down, the prepared LNA probe and quencher mixer can be stored in a refrigerator for up to 7 days. For lncRNA detection, the prepared double-stranded LNA/DNA probe was then transfected into MSCs using Lipofectamine 2000 following manufacturers’ instructions. lncRNA gene expression can thus be evaluated by measuring the fluorescence intensity of MSCs transfected with LNA/DNA probes.

### Simulation of orbital shear stress

To apply the simulated shear, a low-speed orbital shaker (Corning LSE, 6780-FP, orbit) was used. Once MSCs reach 90% confluency, cells were induced using adipocyte induction medium. One plate of cells was then placed on the orbital shaker at the speed of 10 RPM continuously for a total of 10 days. The orbital shaker was placed inside the cell culture incubator to maintain the cell culture environment. The orbital shear stress was calculated using the following equation: $\tau_{\max}=r\times \sqrt{\rho \eta \omega^{3}}$ , where *τ*_*max*_ is near-maximal shear stress, *r* is the orbital radius of rotation, *ρ* is the density of cell culture medium, *η* is the dynamic viscosity of the medium, *ω* is the angular velocity and *ω = 2πf*, where *f* is the frequency of rotation (revolution per second).

### Silencing MALAT1

To silence lncRNA MALAT1 expression, MSCs were seeded in 12-well plates and transfected with MALAT1 antisense probe (Qiagen) at a concentration of 50 nM (final concentration) using Lipofectamine 2000 (Invitrogen) transfection reagent following manufacturer’s instructions. A negative control antisense probe was used as a negative control. After 24 h of transfection, the transfection medium was replaced with a fresh basal culture medium. To investigate the effects of silencing MALAT1 on adipocyte differentiation, induction was initiated after 24 h of MALAT1 silencing.

### Reverse transcription and RT-PCR

To quantify the silencing efficiency of MALAT1 in MSCs, the RT-PCR assay was performed. Initially, cells were seeded in 6-well plates with a concentration of 4 × 10 ^5^ cells/well. After siRNA silencing for 48 h, total RNAs were isolated and reverse-transcribed into cDNA using SuperScript VILO cDNA Syntheis Kit (ThermoFisher, Cat #: 11754050). cDNA samples were then amplified by qPCR. PCR reaction solution was assembly as below: 0.5 µL Taqman Gene Expression Assay (×10) for MALAT1 (Assay ID: Hs00273907_s1, Cat #: 4331182), 5 µL of TaqMan Fast Advanced Master Mix (Cat #:4444556), and 1 µL of cDNA. The total volume is 10 uL. The quantitative PCR was performed on a BioRad Real Time PCR system, and data were collected and analyzed. All samples were prepared and tested in triplicate. The relative expression levels of lncRNAs were determined by equation 2^−ΔΔCt^.

### F-actin staining

The MSCs were washed 3 times with ×1 PBS, 4 min each time. Then the cells were fixed with 4% paraformaldehyde solution (PFA) for 10 min before being permeabilized and blocked with the Perm/Block solution PBST (PBS + 0.5% Triton + 1% BSA) for 1 h. After washing 3 times with ×1 PBS, cells were incubated with phalloidin (1:30) and Hoechst 33342 (1:2,000) for 30 min at room temperature. The cells were then washed 3 times with ×1 PBS before fluorescence imaging and analysis.

### Live/dead viability staining

The viability of MSCs following exposure to orbital shear was assessed using a live/dead viability assay (ThermoFisher). Cells were stained with propidium iodide (PI, 10 μg/mL), a fluorescent agent that binds to DNA by intercalating between base pairs. Hoechst 33,342 was used to stain cell nuclei at a concentration of 20 μM for 30 min. After staining, cells were washed three times with 1x PBS to remove excess dye. Imaging was performed using Texas Red (535/617 nm) and DAPI (360/460 nm) filters on the ZOE imaging station.

### Oil Red O staining

To assess adipocyte differentiation of MSCs, intracellular lipid droplets, which are markers of adipocyte formation during differentiation, were stained using Oil Red O. The working solution were prepared by diluting Oil Red O stock solution 3:2 using deionized H_2_O (diH_2_O) and filtered using filter paper. The staining process were performed based on the manufacturer’s instructions. Briefly, after 5, 7, and 10 days of adipocyte differentiation, MSCs were fixed using 4% PFA for 15 min and then washed 3 times with diH_2_O, followed by incubation of Oil Red O working solution for 15 min at room temperature. MSCs were then washed 5 times with diH_2_O and imaged using bright fields. Positive Oil Red O staining appears as red-stained lipid droplets within the cells, indicating successful adipocyte differentiation. The number of Oil Red-O positive lipid droplets were counted. Droplets with faint colors were ignored.

### Imaging and statistical analysis

Images were obtained using Echo Revolution fluorescent microscope with an integrated digital camera (5 MP CMOS Color for bright field, 5 MP sCMOS Mono for fluorescence imaging). Actin staining images were acquired using a ZOE Fluorescent Cell Imaging Microscope (Bio-Rad). To ensure consistency, all images were captured under identical settings, including exposure time and gain. Image analysis and data collection were performed using NIH ImageJ software. To measure lncRNA MALAT1 expression, the mean fluorescence intensity of each cell was measured, and background noise subtracted. Cells were quantified within the same field of view, with a minimum of five images analyzed for each condition. For actin fiber alignment, we used ImageJ plugin Orientation J to acquire the orientation map and directionality histograms. The experiments were conducted at least three times, with over 100 cells quantified per group. Results were analyzed using independent, two-tailed Student’s t-test in Excel (Microsoft). One-way analysis of variance (ANOVA) was used for multiple group comparisons. P < 0.05 was considered statistically significant.

## Results

### Simulation of orbital shear stress and analysis

To simulate physiologically relevant shear stress and access its effects on adipocyte differentiation, an orbital shaker was utilized to simulate shear stress. We estimated the generated shear using Stokes’ second problem, which describes a plate oscillating along a single axis in the plan of the plate, with liquid above it. Although the shaker does not generate uniform laminar flow on cells, most cells were subjected to near-maximum shear, calculated as: $\tau_{\max}=a\times \sqrt{\rho \eta {\left(2\pi f\right)}^{3}}$ , where *a* is the orbital radius of rotation, *η* and *ρ* are viscosity and density of the culture medium, respectively. We further simulated the distribution of shear stress across the shaker platform using MATLAB to identify the slightly difference of shear stress across the entire platform. As the shaker oscillates along the y-axis, the generated shear stress along this axis remains consistent. The orbital shear was simulated when the shaker was setting at 30 RPM, with maximum shear stress of approximately 0.7 Pa (7.1 dyne/cm^2^) at the edge of the shaker. The well plates were placed on the yellowed labeled region on top of the shaker, Figure 1D. The applied shear stress in different wells ranges from 3 to 7 dyne/cm^2^, which is consistent with the value reported in previous studies (Arora et al., 2020; Knippenberg et al., 2005). This range provides a realistic simulation of the mechanical forces acting on cells in the biological setting.

### Shear stress modulates MSCs morphology and actin organization

**FIGURE 2:**
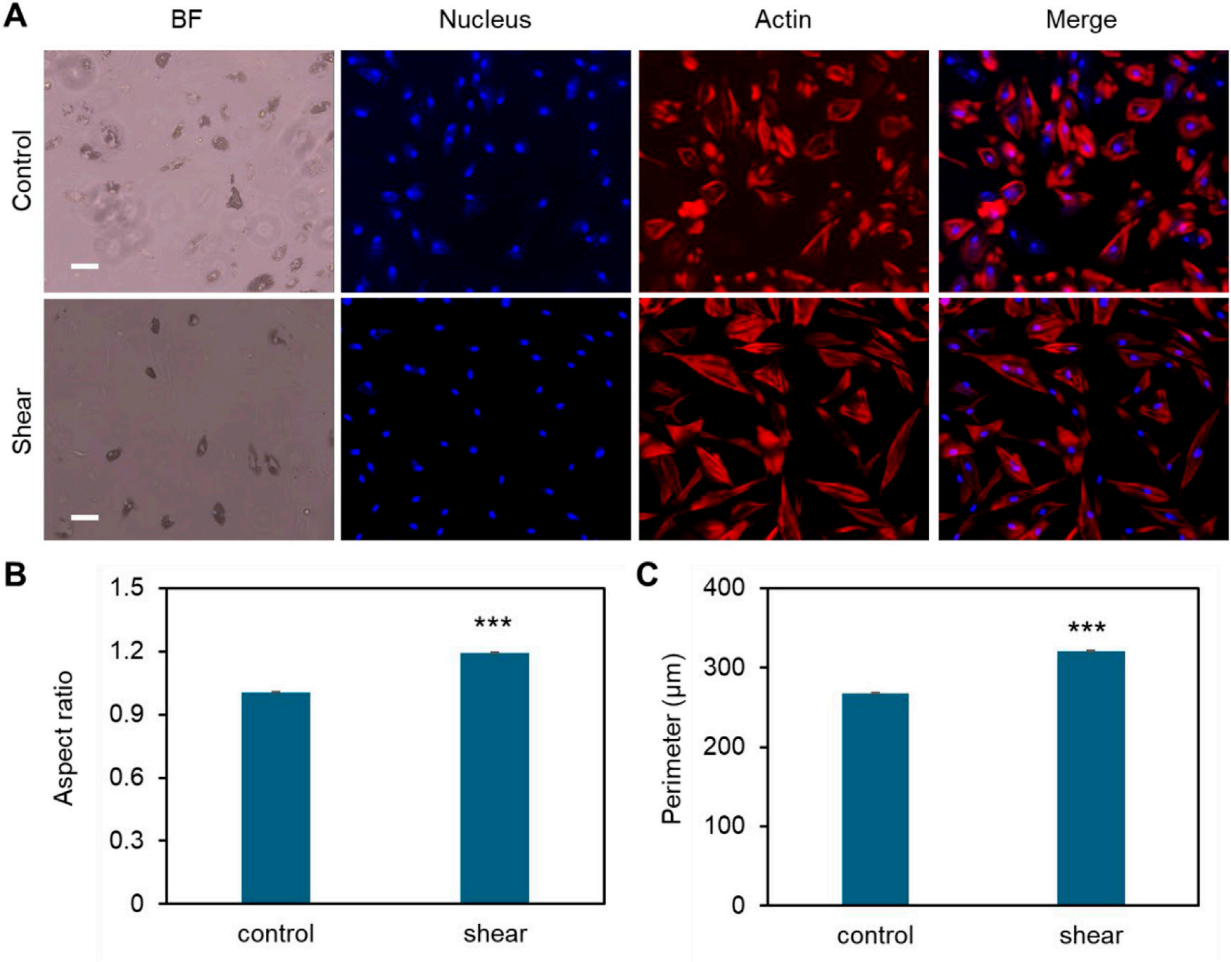
Effects of low fluid shear on MSCs morphology change during adipocyte differentiation. **(A)** Representative bright field and fluorescence images of MSCs under static condition (control) and exposed to shear stress (shear). MSCs were exposed to orbital shear continuously for 5 days. Samples were stained with F-actin (red; by phalloidin), and nuclei (blue; by Hoechst 33342), respectively. Scale bar: 100 μm. **(B)** Comparison of aspect ratio of MSCs after 10 days of exposure to low fluid shear. **(C)** Comparison of observed cell perimeter after 10 days of exposure to shear. Data represents over 200 cells in each group and are expressed as mean ± s.e.m. A two-tailed t-test was used to analyze differences between control and shear conditions. (n = 5, ***, p < 0.001, **, p < 0.01, *, p < 0.05).

**FIGURE 3:**
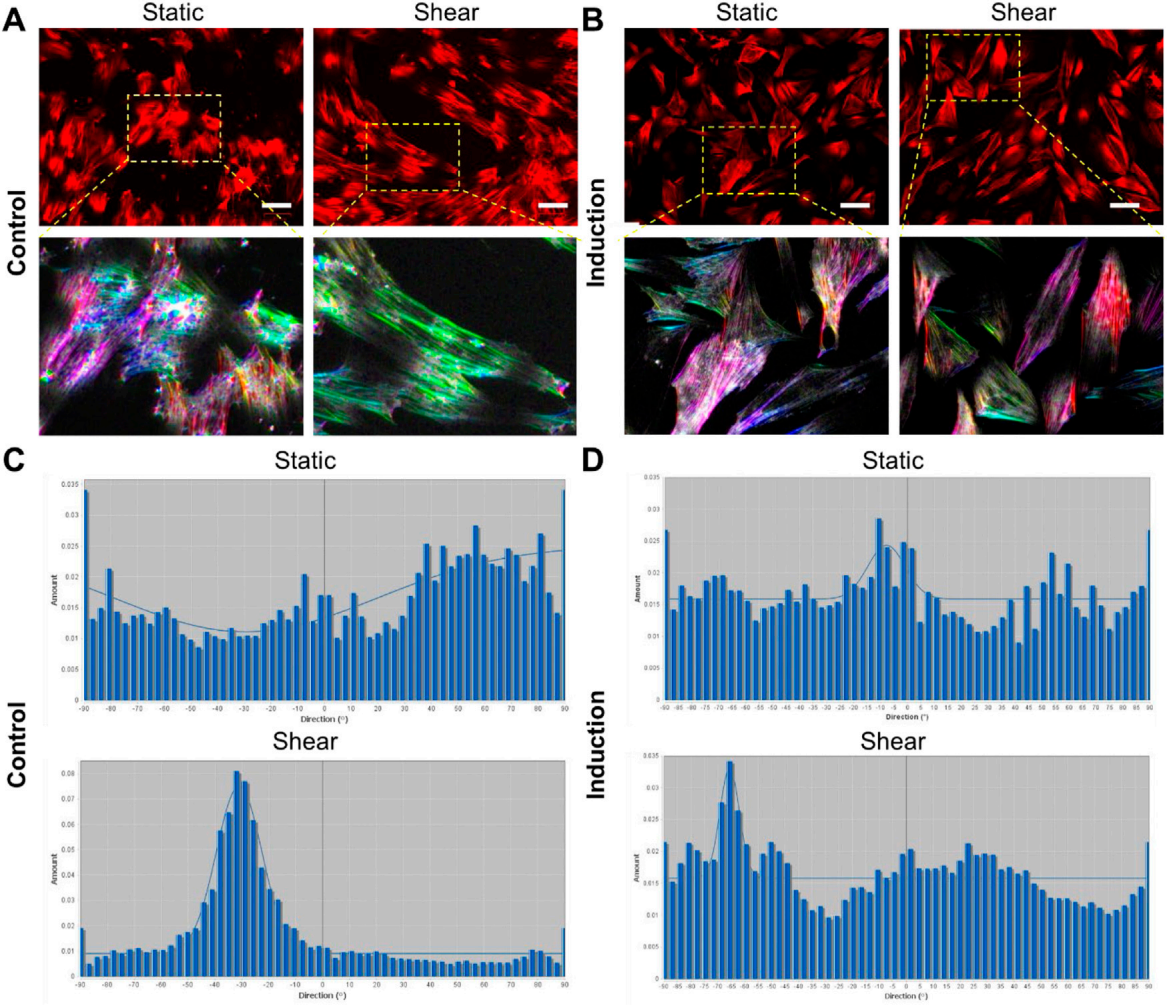
Quantification of actin organization. **(A,B)** Shows representative images of actin distribution under different conditions, without induction **(A)**, and with adipocyte induction **(B)**. Scale bar: 50 µm. The lower panel of **(A,B)** shows enlarged areas of orientational maps. **(A, B)** Top panel: cells were stained with Phalloidin (1:500) for F-actin, bottom panel showed orientational color map of F-actin alignment. Each color indicates a specific direction of aligned F-actin, which provides a visual representation of F-actin orientation inside the cells. More diverse of the color map indicates more random aligned F-actin inside the cell (static condition). Shear stress modified the F-actin distribution with more aligned fibers compared to static condition. **(C, D)** Shows the histogram of directionality of actin alignment with different treatments.

The effects of shear stress on MSCs phenotypic behaviors were first evaluated and compared, including cell viability, cell proliferation, cell aspect ratio, and cell perimeter. The cell viability was assessed using PI/Hoechest 33342 staining assay. Supplementary Figures S1, S2 show the bright field and fluorescent images of MSCs after 5 days and 10 days of culture w/o shear stress, respectively. It is evident that shear stress did not have effects on cell viability, with no dead cells observed in red channel. The effects of shear stress on cell morphology were further assessed by measuring cell aspect ratio and cell perimeter, respectively. Supplementary Figure S3A showed actin cytoskeleton (stained in red), the nuclei (stained in blue), the merged images (actin + nuclei). It is shown that the cells under shear stress (bottom row) appear to have more elongated, aligned actin fibers compared to the control, suggesting that shear stress may influence actin cytoskeleton organization. Quantitative analysis of aspect ratio and perimeter measurements did not show significant differences between the control and shear-exposed cells, suggesting shear stress did not affect the overall cell shape or size in terms of perimeter or aspect ratio. This result suggests that shear stress might influence intracellular organization without drastically changing the overall size or shape of the cells. We next compared cell morphology changes when cells were cultured in adipocyte induction medium. Interestingly, shear stress affects cell aspect ratio and perimeter significantly, Figure 2. Figure 2A showed representative bright field and fluorescence images of MSCs under static and shear condition, respectively. The cell aspect ratio and perimeter of cells that were exposed to shear stress increased significantly, Figures 2B,C. It is noted that actin cytoskeleton is under remodeling when MSCs cultured in adipocyte differentiation medium, Supplementary Figure S4. In the control group, the actin filaments were less organized compared to the cells exposed to shear. However, the actin filaments were still well organized even after 5 days of induction, indicating the strong shear effects on actin organization. We further quantified the actin alignment and compared the difference with and without shear stress, Figure 3. With shear stress, the actin structure was more aligned compared with static condition, for both control and adipocyte induction groups. Orientational maps further confirmed the actin alignment, Figures 3A,B lower panel. The directionality of actin structure was further quantified and compared, Figures 3C,D. Without adipocyte induction, the actin fibers was distributed randomly at static condition, shear stress changed the fiber alignment, with more fibers aligned with the direction of the shear. The normal distribution of histogram of actin fibers alignment indicates that the fiber mainly aligned in one direction (Figure 3C lower panel). For adipocyte differentiation groups, less actin fibers were observed for both static and shear conditions. The histogram shows that actin fibers were randomly distributed inside the cells at static condition. Actin fibers in shear treated cells are more aligned compared to the static condition.

### Shear stress inhibits adipocyte differentiation

**FIGURE 4:**
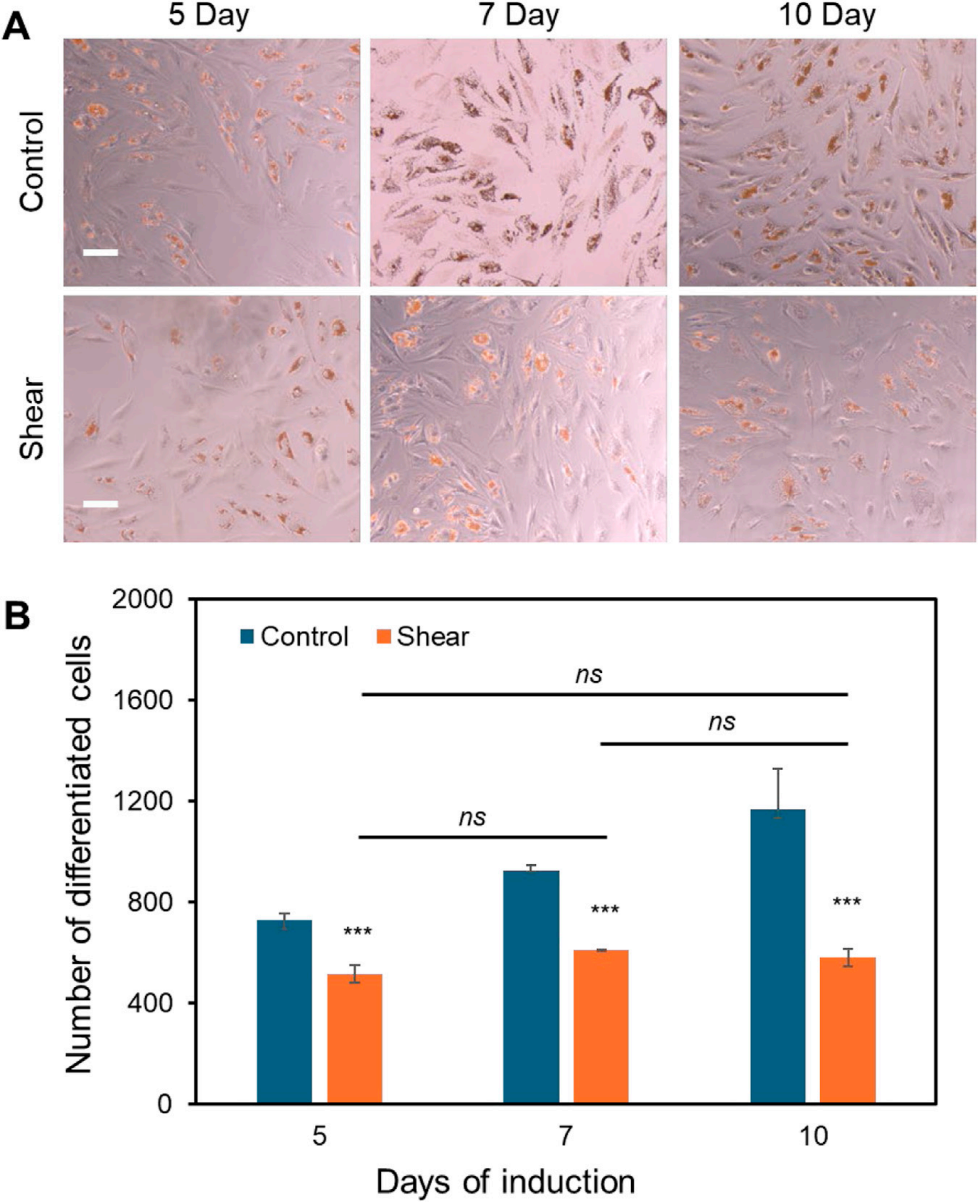
Low fluid shear inhibits adipocyte differentiation. **(A)** Representative images of adipocyte differentiation after 5, 7, and 10 days with and without shear. **(B)** Quantitative results of adipocyte comparison. The differentiated adipocytes were quantified and compared by measuring the number of lipid droplets at each field of view. Field of view: 2,172 μm × 1,817 µm. Data represents over 500 cells in each group and are expressed as mean ± s.e.m. One-way analysis of variance (ANOVA) was used for multiple group comparisons. A two-tailed t-test was used to analyze differences between control and shear conditions. (n = 5, ***, p < 0.001, **, p < 0.01, *, p < 0.05).

Previously, we have reported that low fluid shear stress enhances MSCs osteogenic differentiation with enhanced ALP enzyme activity (Zhao et al., 2022a), however, it is obscure how shear affects adipocyte differentiation. Thus, the effects of shear stress on MSCs adipocyte differentiation were further elucidated and compared. Briefly, cells were initially seeded in two well plates and cultured in basal medium under static condition. Once the cells reached 70%–80% confluency, basal medium was replaced by induction medium for both plates. Meanwhile, one well plate was placed on top of the orbital shaker, while the other plate was placed in static condition without exposure to shear. After 5, 7, and 10 days of induction and shaking, adipocyte differentiation was evaluated and compared by measuring oil-red-o-stained cells, a biochemical marker for lipid accumulation, a crucial step for adipocyte formation. Figure 4A showed the representative MSCs images after 5, 7 and 10 days of adipocyte induction in control and shear condition, respectively. We further quantified the differentiated cells by counting the oil-red-o-stained adipocyte lipid droplets in each field of view. Figure 4B showed comparison of differentiated cells at different time points, with and without shear stress. It is evident that for the cells without shear stress, the number of differentiated cells increases as the days increase. However, for the cells exposed to shear stress, the number of differentiated cells was significantly decreased, and the number was not increased after 7 and 10 days of induction. Together with the previous observation, the results indicate that shear stress modulates cell morphology, proliferation, and inhibits adipocyte differentiation.

### Shear stress modulates the expression of MALAT1

**FIGURE 5:**
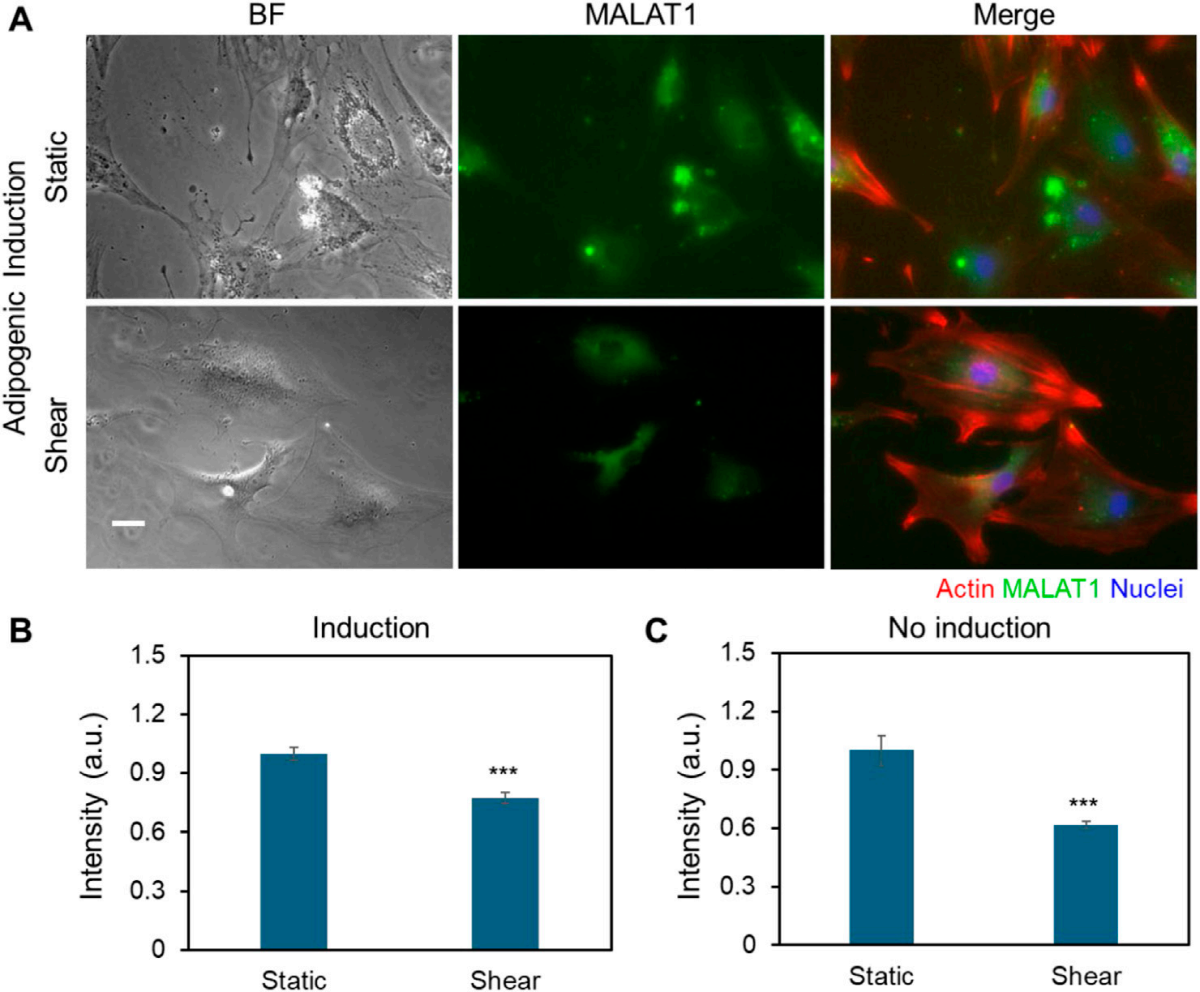
Shear stress modulates the expression of MALAT1. **(A)** Representative bright field and fluorescence images of MSCs in control and shear conditions with adipogenic induction medium (5 days), respectively. The green fluorescence signal shows MALAT1 expression. Red: Phalloidin (F-actin), blue: Hoechst 33342 (Nucleus). Scale bar: 100 µm. **(B)** Comparison of mean fluorescence intensity of MALAT1 expression for MSCs cultured in adipogenic induction medium. **(C)** Comparison of mean fluorescence intensity of MALAT1 expression for MSCs cultured in basal medium. Data are expressed as mean ± s.e.m. (n = 3). A two-tailed t-test was used to analyze differences between control and shear conditions. *, p < 0.05; **, p < 0.01; ***, p < 0.005.

Previous studies indicate that lncRNAs are responsive to biophysical cues, indicating their mechanosensitive roles in regulating cell functions, including proliferation, migration, and differentiation. Wu et al. demonstrated that lncRNA H19 promotes osteogenic differentiation by acting as a molecular sponge for miR-135, thereby modulating mesenchymal stem cell (MSC) activity through FAK signaling in a tension-dependent manner (Wu et al., 2018). A new lncRNA that is related to mechanical stress (lncRNA-MSR) would hijack miR-152 to control TMSB4 expression in cyclic tensile strain-induced regulation of chondrocytes and enhance cartilage degradation (Liu et al., 2016). Thus, to test whether MALAT1 is responsive to shear stress, we utilized an LNA/DNA gapmer nanobiosensor to detect MALAT1 expression in MSCs (Fasciano et al., 2023). Figure 5A showed the bright field and fluorescence images of MSCs under control and shear in adipogenic induction medium, respectively. To better understand the correlation of cytoskeleton organization and MALAT1 expression, we further stained F-actin. It is evident that in both static and shear condition, cells with well-aligned actin fibers have low expression of MALAT1. Cells under shear stress have more aligned actin structure compared to cells under static condition. The results showed that shear stress inhibits MALAT1 expression with reduced fluorescence signal. We further quantified the inhibition effects by measuring the mean fluorescence intensity of each individual cells and compared, Figure 5B. We also compared and quantified the expression of MSCs without induction under static and shear condition (Figure 5C; Supplementary Figure S5). For the cells exposed to shear stress, the expression of lncRNA MALAT1 was reduced by approximately 30% compared to the cells without shear. Taken together, the results shown MALAT1 is responsive to mechanical shear stress, indicating its mechanosensitive role in stem cell fate determination.

### MALAT1 knockdown inhibits adipocyte differentiation of MSCs

**FIGURE 6:**
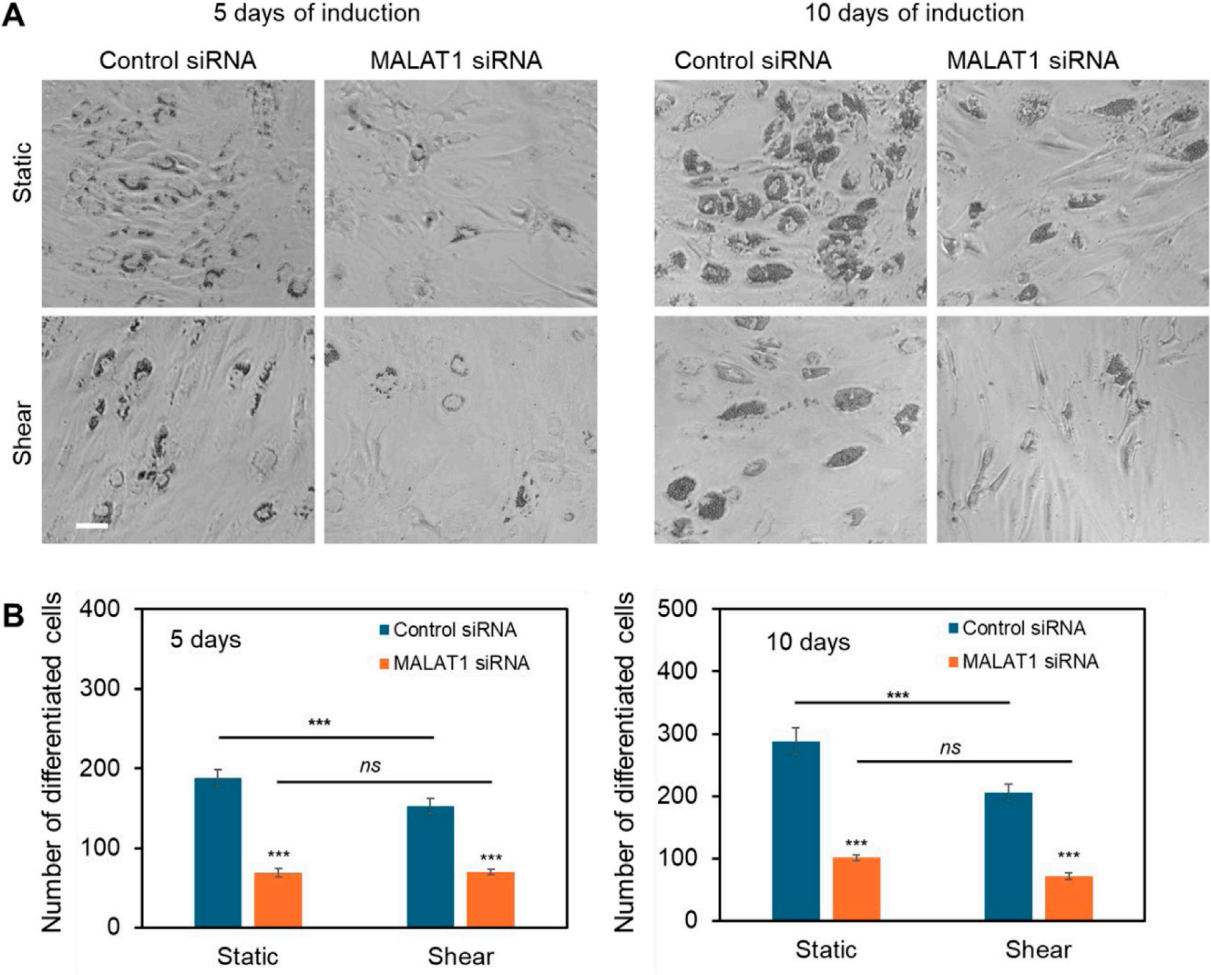
lncRNA MALAT1 knockdown inhibits adipocyte differentiation. **(A)** Representative images of differentiated MSCs in different conditions (static and shear) after 5 and 10 days of induction. Scale bar: 100 µm. **(B)** Comparison of the number of differentiated cells after MALAT1 silencing for both cells under static and shear conditions (n = 3). Data are expressed as mean ± s.e.m. (n = 3). One-way analysis of variance (ANOVA) was used for multiple group comparisons. A two-tailed t-test was used to analyze differences between control and shear conditions. *, p < 0.05; **, p < 0.01; ***, p < 0.005.

It had been reported that lncRNA MALAT1 enhances osteogenic differentiation of bone-derived and adipose-derived MSCs through either sponging to miRNAs by enhancing downstream genes expression, including Runx2, ALP, and BMP (Gao et al., 2018). Our group also showed earlier MALAT1 enhance osteogenic differentiation with increased ALP activity and calcium deposition. However, the role of MALAT1 in adipocyte differentiation was not well explored, especially the crosstalk of MALAT1 and biophysical cues. Thus, we investigated the role of MALAT1 in adipocyte differentiation and the effects of shear stress. We first silenced MALAT1 using siRNA knockdown. A negative control siRNA was used as a control for comparison. Briefly, cells were seeded in well plates and silenced using control siRNA and MALAT1 siRNA, following manufacture’s instructions. The silencing efficiency was compared, Supplementary Figure S6. To evaluate the effects of shear stress on MALAT1 silenced cells, the cells were placed on the shaker for the duration of adipogenic induction. Figure 6A showed representative images of differentiated MSCs in different conditions after 5 and 10 days of induction. The results showed silencing MALAT1 inhibits adipocyte differentiation with reduced lipid droplets and nodules after 5- and 10- days induction. Without applying shear, MALAT1 siRNA knockdown reduced the number of differentiated cells by 62% and 65% after 5 and 10 days of induction, respectively (Figure 6B). For the cells exposed to shear stress, MALAT1 knockdown reduced adipocyte differentiation by 54% and 65% after 5- and 10- days of induction, respectively (Figure 6B). It is noted that applying shear stress did not further inhibit adipocyte differentiation for the MALAT1 siRNA silenced groups. This result indicates that shear stress may downregulate the expression of MALAT1, leading to a reduction in lipid droplet accumulation within cells.

## Discussion

Biophysical and biochemical cues are key parameters that regulate cell behaviors, including proliferation, motility, and differentiation. Emerging evidence has shown that biophysical cues, including shear, stretch, and stiffness is pivotal in directing stem cell fate determination. Although numerous studies have shown that stem cells are mechanosensitive and respond to mechanical stimuli, it is obscure which genome is directly responsive the stimulation. In this study, we first investigated the effects of shear stress on MSCs behavior, including cell proliferation and adipocyte differentiation. We next studied the role of lncRNA MALAT1 in MSCs differentiation and elucidated the correlation between shear stress and the expression of MALAT1 in MSCs. We discovered that MALAT1 expression was downregulated when cells were exposed to shear stress, indicating its mechanosensitive role. By utilizing a novel gapmer dsLNA nanobiosensor, we detected and compared MALAT1 expression in MSCs. Previous studies have shown that this dsLNA nanobiosensor can track spatiotemporal RNA dynamics in cell migration (Riahi et al., 2013), mice lung cancer (Riahi et al., 2014), wounded corneal tissue repair (Wang et al., 2015), liver tissue (Tao et al., 2014), synthetic cells (Fasciano and Wang, 2023; Wang S. et al., 2018), and vasculature formation (Wang et al., 2016; Wang et al., 2017). This nanobiosensor was also utilized for lncRNA, miRNA, and mRNA detection in stem cells. (Zhao et al., 2022a; Zhao et al., 2022b; Fasciano et al., 2023; Wang S. et al., 2018; Dean et al., 2015; Zhao and Wang, 2023; Fasciano et al., 2023; Zhao and Wang, 2023; Dean et al., 2016).

Adipocyte differentiation plays a crucial role in bone remodeling because adipocytes within bone marrow secrete various factors that can directly modulate the activity of osteoblasts and osteoclast, thus affecting overall bone mass and skeletal integrity (Pachón-Peña and Bredella, 2022). Imbalanced adipocyte differentiation and osteoblast differentiation within bone marrow microenvironment could potentially lead to bone diseases. Excessive adipocyte differentiation can lead to decreased bone mass, contributing to conditions like osteoporosis, the most common bone remodeling disorder worldwide (Tencerova et al., 2021; Liu et al., 2023). For example, increased marrow fat content has been observed in most bone loss conditions. In addition, bone marrow adipose tissue alterations have been associated with systemic conditions like anemia (Wang H. et al., 2018), glucose intolerance, and cancer (de Paula and Rosen, 2020). It was also reported that exposure to space with removed physical forces causes a 10-fold increase in adipocyte differentiation in animal models (Liu et al., 2023). The tightly controlled lineage commitment of MSCs plays a critical role in maintaining bone homeostasis, which is essential for proper function and balance of bone tissue. Therefore, understanding lineage commitment of MSCs to adipocyte is essential and could provide effective therapeutic regime for related bone diseases. In bone marrow microenvironment, bone cells sense mechanical load in response to fluid shear stress, initiating a signal for cellular excitation (Weinbaum et al., 1994). The fluid flow is critical for mechanotransduction as well as enhancing convective solute transport within the bone (Price et al., 2011). Thus, we investigated the effects of fluid shear affects MSCs adipocyte differentiation. Our results showed that in a simulated physiological shear environment, adipocyte differentiation is inhibited. Taking together with our previous finding that shear stress enhance osteogenic differentiation (Zhao et al., 2022a), these results indicate that bone marrow origin of MSCs has the potential to differentiate into both osteoblast and adipocyte and maintain a balance between them.

MALAT1, one of the few highly conserved nuclear noncoding RNA, is abundantly expressed in normal tissues. MALAT1 has been shown to regulate gene expression at transcriptional and post-transcriptional levels, modulating chromatin organization, splicing, and mRNA stability. Initially, MALAT1 was indicated in tumorigenesis, metastasis, and cardiovascular diseases. Recently, emerging evidence showed MALAT1 plays a crucial role in bone remodeling and bone related diseases. Zhao et al. reported that MALAT1 deficiency promotes osteoporosis and bone metastasis of melanoma, thus they concluded that MALAT1 protects against osteoporosis and bone metastasis (Zhao et al., 2024). Several studies also showed low expression of MALAT1 is related to reduced osteoblast differentiation and cause bone mass loss (Liu et al., 2024; Jiang et al., 2024). Nevertheless, how MALAT1 responses to mechanical cues, i.e., shear stress, has not been explored. Based on literature studies and our previous findings, we hypothesize that MALAT1 is a mechanosensitive biomarker that regulates bone remodeling by controlling osteogenic and adipocyte differentiation. We further verified that MALAT1 expression was downregulated in MSCs that were exposed to shear stress. Without shear stress MSCs were induced to adipocyte differentiation, which is consistent with previous findings that reduced shear stress or altered shear stress promote adipocyte differentiation, which eventually causes reduced osteoblast differentiation, thus altering the bone homeostasis.

The differentiation of MSCs is a two-step process that include lineage commitment, which transitions MSCs into lineage-specific progenitors, and maturation, evolving progenitors into specific cell types. Several key regulators have been identified in regulating MSCs lineage commitment, including transforming growth factor-beta (TGFβ)/Bone morphogenetic protein (BMP) signaling (Chen et al., 2012), wingless-type MMTV integration site (Wnt) signaling (Hu et al., 2024), Hedgehog (Hh) (Yang et al., 2015), and Notch signaling (Dishowitz et al., 2012; Stellpflug et al., 2025). Although our study demonstrates the involvement of MALAT1 in shear stress-mediated inhibition of adipogenesis, the underlying upstream and downstream signaling pathways remain unclear. Specifically, MALAT1 may modulate adipocyte differentiation by acting as a molecular sponge for microRNAs, thereby influencing downstream gene expression profiles. Furthermore, shear stress-induced suppression of MALAT1 expression and adipogenesis may involve alterations in cytoskeletal organization, a critical factor in determining stem cell lineage commitment. Future research should elucidate the mechanistic connection between cytoskeletal dynamics and MALAT1 regulation by examining shear stress-induced changes in Rac1 and RhoA signaling activities and their effects on actin remodeling and adipocyte differentiation.

## Conclusion

In this study, we aimed to identify the mechanosensitive role of lncRNA MALAT1 during MSCs adipocyte differentiation. We first evaluated the physiological relevant shear stress on cell behaviors including morphological changes and differentiation capacity. Our results showed that cell viability and cell morphology were not affected by shear stress. However, once MSCs went through adipocyte differentiation, cells exposed shear showed elongated morphology with increased cell aspect ratio and perimeters. Interestingly, shear stress inhibits adipocyte differentiation with reduced lipid droplets and nodules. By disrupting the expression of lncRNA MALAT1, we further assessed the role of MALAT1 in regulating adipocyte differentiation. We showed silencing MALAT1 significantly mediated adipocyte differentiation. Thus, it is intriguing to study how shear stress modulates MALAT1 expression. By utilizing a novel gapmer dsLNA nanobiosensor, we monitored MALAT1 expression in MSCs and discovered that shear stress inhibits MALAT1 expression with significantly reduced fluorescence intensity. Quantitative RT-PCR analysis further validated this downregulation. Taken together, we conclude that shear stress inhibits MSCs adipocyte differentiation through downregulation of lncRNA MALAT1, indicating the crosstalk between biophysical cues and lncRNAs.

## Data Availability

The original contributions presented in the study are included in the article/Supplementary Material, further inquiries can be directed to the corresponding author.

## References

[B1] Adeniran-Catlett A. E. Weinstock L. D. Bozal F. K. Beguin E. Caraballo A. T. Murthy S. K. (2016). Accelerated adipogenic differentiation of h MSC s in a microfluidic shear stimulation platform. Biotechnol. Prog. 32 (2), 440–446. 10.1002/btpr.2211 26587686

[B2] Aich M. Chakraborty D. (2020). Role of lncRNAs in stem cell maintenance and differentiation. Curr. Top. Dev. Biol. 138, 73–112. 10.1016/bs.ctdb.2019.11.003 32220299

[B3] Arora S. Srinivasan A. Leung C. M. Toh Y.-C. (2020). Bio-mimicking shear stress environments for enhancing mesenchymal stem cell differentiation. Curr. Stem Cell Res. and Ther. 15 (5), 414–427. 10.2174/1574888x15666200408113630 32268869

[B4] Chen G. Deng C. Li Y.-P. (2012). TGF-β and BMP signaling in osteoblast differentiation and bone formation. Int. J. Biol. Sci. 8 (2), 272–288. 10.7150/ijbs.2929 22298955 PMC3269610

[B5] Choi J. Lee S. Y. Yoo Y.-M. Kim C. H. (2017). Maturation of adipocytes is suppressed by fluid shear stress. Cell Biochem. Biophysics 75, 87–94. 10.1007/s12013-016-0771-4 27830366

[B6] Dean Z. S. Elias P. Jamilpour N. Utzinger U. Wong P. K. (2016). Probing 3D collective cancer invasion using double-stranded locked nucleic acid biosensors. Anal. Chem. 88 (17), 8902–8907. 10.1021/acs.analchem.6b02608 27529634 PMC5488859

[B7] Dean Z. S. Riahi R. Wong P. K. (2015). Spatiotemporal dynamics of microRNA during epithelial collective cell migration. Biomaterials 37, 156–163. 10.1016/j.biomaterials.2014.10.022 25453946 PMC4312218

[B8] de Paula F. J. Rosen C. J. (2020). Marrow adipocytes: origin, structure, and function. Annu. Rev. physiology 82 (1), 461–484. 10.1146/annurev-physiol-021119-034513 31702948

[B9] Dishowitz M. I. Terkhorn S. P. Bostic S. A. Hankenson K. D. (2012). Notch signaling components are upregulated during both endochondral and intramembranous bone regeneration. J. Orthop. Res. 30 (2), 296–303. 10.1002/jor.21518 21818769 PMC3210892

[B10] Fahy N. Alini M. Stoddart M. J. (2018). Mechanical stimulation of mesenchymal stem cells: implications for cartilage tissue engineering. J. Orthop. Res. 36 (1), 52–63. 10.1002/jor.23670 28763118

[B11] Fasciano S. Luo S. Wang S. (2023). Long non-coding RNA (lncRNA) MALAT1 in regulating osteogenic and adipogenic differentiation using a double-stranded gapmer locked nucleic acid nanobiosensor. Analyst 148 (24), 6261–6273. 10.1039/d3an01531a 37937546

[B12] Fasciano S. Wang S. (2023). Recent advances of droplet-based microfluidics for engineering artificial cells. SLAS Technol. 29 (2), 100090. 10.1016/j.slast.2023.05.002 37245659

[B13] Fernandes M. Valente S. G. Sabongi R. G. Dos Santos J. B. G. Leite V. M. Ulrich H. (2018). Bone marrow-derived mesenchymal stem cells versus adipose-derived mesenchymal stem cells for peripheral nerve regeneration. Neural Regen. Res. 13 (1), 100. 10.4103/1673-5374.224378 29451213 PMC5840974

[B14] Fitzsimmons R. E. Mazurek M. S. Soos A. Simmons C. A. (2018). Mesenchymal stromal/stem cells in regenerative medicine and tissue engineering. Stem cells Int. 2018 (1), 1–16. 10.1155/2018/8031718 PMC6120267 30210552

[B15] Gao Y. Xiao F. Wang C. Wang C. Cui P. Zhang X. (2018). Long noncoding RNA MALAT1 promotes osterix expression to regulate osteogenic differentiation by targeting miRNA-143 in human bone marrow-derived mesenchymal stem cells. J. Cell. Biochem. 119 (8), 6986–6996. 10.1002/jcb.26907 29741283

[B16] Guilak F. Cohen D. M. Estes B. T. Gimble J. M. Liedtke W. Chen C. S. (2009). Control of stem cell fate by physical interactions with the extracellular matrix. Cell stem cell 5 (1), 17–26. 10.1016/j.stem.2009.06.016 19570510 PMC2768283

[B17] Han Y. Li X. Zhang Y. Han Y. Chang F. Ding J. (2019). Mesenchymal stem cells for regenerative medicine. Cells 8 (8), 886. 10.3390/cells8080886 31412678 PMC6721852

[B18] Heo S.-J. Driscoll T. P. Thorpe S. D. Nerurkar N. L. Baker B. M. Yang M. T. (2016). Differentiation alters stem cell nuclear architecture, mechanics, and mechano-sensitivity. Elife 5, e18207. 10.7554/elife.18207 27901466 PMC5148611

[B19] Hu L. Chen W. Qian A. Li Y.-P. (2024). Wnt/β-catenin signaling components and mechanisms in bone formation, homeostasis, and disease. Bone Res. 12 (1), 39. 10.1038/s41413-024-00342-8 38987555 PMC11237130

[B20] Jiang C. Wang P. Tan Z. Zhang Y. (2024). Long non-coding RNAs in bone formation: key regulators and therapeutic prospects. Open Life Sci. 19 (1), 20220908. 10.1515/biol-2022-0908 39156986 PMC11330173

[B21] Ju C. Liu R. Zhang Y.-W. Zhang Y. Zhou R. Sun J. (2019). Mesenchymal stem cell-associated lncRNA in osteogenic differentiation. Biomed. and Pharmacother. 115, 108912. 10.1016/j.biopha.2019.108912 31048188

[B23] Knippenberg M. Helder M. N. Zandieh Doulabi B. Semeins C. M. Wuisman P. I. Klein-Nulend J. (2005). Adipose tissue-derived mesenchymal stem cells acquire bone cell-like responsiveness to fluid shear stress on osteogenic stimulation. Tissue Eng. 11 (11-12), 1780–1788. 10.1089/ten.2005.11.1780 16411823

[B24] Lanzillotti C. De Mattei M. Mazziotta C. Taraballi F. Rotondo J. C. Tognon M. (2021). Long non-coding RNAs and microRNAs interplay in osteogenic differentiation of mesenchymal stem cells. Front. Cell Dev. Biol. 9, 646032. 10.3389/fcell.2021.646032 33898434 PMC8063120

[B25] Leach J. K. Whitehead J. (2017). Materials-directed differentiation of mesenchymal stem cells for tissue engineering and regeneration. ACS biomaterials Sci. and Eng. 4 (4), 1115–1127. 10.1021/acsbiomaterials.6b00741 PMC6052883 30035212

[B26] Li B. Luan S. Chen J. Zhou Y. Wang T. Li Z. (2020). The MSC-derived exosomal lncRNA H19 promotes wound healing in diabetic foot ulcers by upregulating PTEN via microRNA-152-3p. Mol. Therapy-Nucleic Acids 19, 814–826. 10.1016/j.omtn.2019.11.034 PMC7005423 31958697

[B27] Li J. Meng H. Bai Y. Wang K. (2016). Regulation of lncRNA and its role in cancer metastasis. Oncol. Res. 23 (5), 205–217. 10.3727/096504016x14549667334007 27098144 PMC7838649

[B28] Li M. Xie Z. Wang P. Li J. Liu W. Tang S. a. (2018). The long noncoding RNA GAS5 negatively regulates the adipogenic differentiation of MSCs by modulating the miR-18a/CTGF axis as a ceRNA. Cell Death and Dis. 9 (5), 554. 10.1038/s41419-018-0627-5 PMC5945827 29748618

[B29] Li R. Zhang W. Yan Z. Liu W. Fan J. Feng Y. (2021). Long non-coding RNA (LncRNA) HOTAIR regulates BMP9-induced osteogenic differentiation by targeting the proliferation of mesenchymal stem cells (MSCs). Aging (Albany NY) 13 (3), 4199–4214. 10.18632/aging.202384 33461171 PMC7906180

[B30] Li S.-N. Wu J.-F. (2020). TGF-β/SMAD signaling regulation of mesenchymal stem cells in adipocyte commitment. Stem Cell Res. and Ther. 11 (1), 41. 10.1186/s13287-020-1552-y 31996252 PMC6990519

[B31] Lin H. Sohn J. Shen H. Langhans M. T. Tuan R. S. (2019). Bone marrow mesenchymal stem cells: aging and tissue engineering applications to enhance bone healing. Biomaterials 203, 96–110. 10.1016/j.biomaterials.2018.06.026 29980291 PMC6733253

[B32] Liu Q. Hu X. Zhang X. Dai L. Duan X. Zhou C. (2016). The TMSB4 pseudogene LncRNA functions as a competing endogenous RNA to promote cartilage degradation in human osteoarthritis. Mol. Ther. 24 (10), 1726–1733. 10.1038/mt.2016.151 27469625 PMC5112043

[B33] Liu T. Melkus G. Ramsay T. Sheikh A. Laneuville O. Trudel G. (2023). Bone marrow adiposity modulation after long duration spaceflight in astronauts. Nat. Commun. 14 (1), 4799. 10.1038/s41467-023-40572-8 37558686 PMC10412640

[B34] Liu W. Zhang Y. Li Q. Wang X. Wu Y. Shen H. (2024). Advances of long non-coding RNAs in osteoclast differentiation and osteoporosis. Pathology-Research Pract. 260, 155413. 10.1016/j.prp.2024.155413 38981344

[B35] Pachón-Peña G. Bredella M. A. (2022). Bone marrow adipose tissue in metabolic health. Trends Endocrinol. and Metabolism 33 (6), 401–408. 10.1016/j.tem.2022.03.003 PMC9098665 35396163

[B36] Pittenger M. F. Discher D. E. Péault B. M. Phinney D. G. Hare J. M. Caplan A. I. (2019). Mesenchymal stem cell perspective: cell biology to clinical progress. NPJ Regen. Med. 4 (1), 22–15. 10.1038/s41536-019-0083-6 31815001 PMC6889290

[B37] Potolitsyna E. Hazell Pickering S. Germier T. Collas P. Briand N. (2022). Long non-coding RNA HOTAIR regulates cytoskeleton remodeling and lipid storage capacity during adipogenesis. Sci. Rep. 12 (1), 10157. 10.1038/s41598-022-14296-6 35710716 PMC9203762

[B38] Price C. Zhou X. Li W. Wang L. (2011). Real-time measurement of solute transport within the lacunar-canalicular system of mechanically loaded bone: direct evidence for load-induced fluid flow. J. Bone Mineral Res. 26 (2), 277–285. 10.1002/jbmr.211 PMC3179346 20715178

[B39] Riahi R. Dean Z. Wu T.-H. Teitell M. A. Chiou P.-Y. Zhang D. D. (2013). Detection of mRNA in living cells by double-stranded locked nucleic acid probes. Analyst 138 (17), 4777–4785. 10.1039/c3an00722g 23772441 PMC3736730

[B40] Riahi R. Wang S. Long M. Li N. Chiou P.-Y. Zhang D. D. (2014). Mapping photothermally induced gene expression in living cells and tissues by nanorod-locked nucleic acid complexes. ACS Nano 8 (4), 3597–3605. 10.1021/nn500107g 24645754 PMC4004321

[B41] Stellpflug A. Caron J. Fasciano S. Wang B. Wang S. (2025). Bone-derived nanoparticles (BNPs) enhance osteogenic differentiation via Notch signaling. Nanoscale Adv. 7, 735–747. 10.1039/d4na00797b 39823045 PMC11734751

[B42] Tao S. Wang S. Moghaddam S. J. Ooi A. Chapman E. Wong P. K. (2014). Oncogenic KRAS confers chemoresistance by upregulating NRF2. Cancer Res. 74 (24), 7430–7441. 10.1158/0008-5472.can-14-1439 25339352 PMC4268230

[B43] Tencerova M. Ferencakova M. Kassem M. (2021). Bone marrow adipose tissue: role in bone remodeling and energy metabolism. Best Pract. and Res. Clin. Endocrinol. and Metabolism 35 (4), 101545. 10.1016/j.beem.2021.101545 33966979

[B44] Wang H. Leng Y. Gong Y. (2018b). Bone marrow fat and hematopoiesis. Front. Endocrinol. 9, 694. 10.3389/fendo.2018.00694 PMC6280186 30546345

[B45] Wang S. Majumder S. Emery N. J. Liu A. P. (2018a). Simultaneous monitoring of transcription and translation in mammalian cell-free expression in bulk and in cell-sized droplets. Synth. Biol. 3 (1), ysy005. 10.1093/synbio/ysy005 PMC6034425 30003145

[B46] Wang S. Riahi R. Li N. Zhang D. D. Wong P. K. (2015). Single cell nanobiosensors for dynamic gene expression profiling in native tissue microenvironments. Adv. Mater. 27 (39), 6034–6038. 10.1002/adma.201502814 26314800

[B47] Wang S. Sun J. Xiao Y. Lu Y. Zhang D. D. Wong P. K. (2017). Intercellular tension negatively regulates angiogenic sprouting of endothelial tip cells via notch1-dll4 signaling. Adv. Biosyst. 1 (1-2), 1600019. 10.1002/adbi.201600019 30662935 PMC6338428

[B48] Wang S. Sun J. Zhang D. Wong P. (2016). A nanobiosensor for dynamic single cell analysis during microvascular self-organization. Nanoscale 8 (38), 16894–16901. 10.1039/c6nr03907c 27547924 PMC5042875

[B49] Wang Y. K. Chen C. S. (2013). Cell adhesion and mechanical stimulation in the regulation of mesenchymal stem cell differentiation. J. Cell. Mol. Med. 17 (7), 823–832. 10.1111/jcmm.12061 23672518 PMC3741348

[B50] Wang Y.-K. Yu X. Cohen D. M. Wozniak M. A. Yang M. T. Gao L. (2012). Bone morphogenetic protein-2-induced signaling and osteogenesis is regulated by cell shape, RhoA/ROCK, and cytoskeletal tension. Stem cells Dev. 21 (7), 1176–1186. 10.1089/scd.2011.0293 21967638 PMC3328763

[B51] Weinbaum S. Cowin S. C. Zeng Y. (1994). A model for the excitation of osteocytes by mechanical loading-induced bone fluid shear stresses. J. biomechanics 27 (3), 339–360. 10.1016/0021-9290(94)90010-8 8051194

[B52] Wiewiora M. Piecuch J. Glűck M. Slowinska-Lozynska L. Sosada K. (2013). Shear stress and flow dynamics of the femoral vein among obese patients who qualify for bariatric surgery. Clin. Hemorheol. Microcirc. 54 (3), 313–323. 10.3233/ch-131736 23686091

[B53] Wu H.-W. Lin C.-C. Hwang S.-M. Chang Y.-J. Lee G.-B. (2011). A microfluidic device for chemical and mechanical stimulation of mesenchymal stem cells. Microfluid. Nanofluidics 11, 545–556. 10.1007/s10404-011-0820-7

[B54] Wu J. Zhao J. Sun L. Pan Y. Wang H. Zhang W.-B. (2018). Long non-coding RNA H19 mediates mechanical tension-induced osteogenesis of bone marrow mesenchymal stem cells via FAK by sponging miR-138. Bone 108, 62–70. 10.1016/j.bone.2017.12.013 29253550

[B55] Xue X. Hong X. Fu J. Deng C. (2016). Regulation of cytoskeleton contractility and osteogenesis of human mesenchymal stem cells using acoustic tweezing cytometry (ATC). Biophysical J. 110 (3), 134a. 10.1016/j.bpj.2015.11.768

[B56] Xue X. Hong X. Li Z. Deng C. X. Fu J. (2017). Acoustic tweezing cytometry enhances osteogenesis of human mesenchymal stem cells through cytoskeletal contractility and YAP activation. Biomaterials 134, 22–30. 10.1016/j.biomaterials.2017.04.039 28453955 PMC5506541

[B57] Yan L. Yang M. Guo H. Yang L. Wu J. Li R. (2013). Single-cell RNA-Seq profiling of human preimplantation embryos and embryonic stem cells. Nat. Struct. and Mol. Biol. 20 (9), 1131–1139. 10.1038/nsmb.2660 23934149

[B58] Yang J. Andre P. Ye L. Yang Y.-Z. (2015). The Hedgehog signalling pathway in bone formation. Int. J. oral Sci. 7 (2), 73–79. 10.1038/ijos.2015.14 26023726 PMC4817553

[B59] Yi J. Liu D. Xiao J. (2019). LncRNA MALAT1 sponges miR-30 to promote osteoblast differentiation of adipose-derived mesenchymal stem cells by promotion of Runx2 expression. Cell tissue Res. 376 (1), 113–121. 10.1007/s00441-018-2963-2 30511267

[B60] Zhang N. Hu X. He S. Ding W. Wang F. Zhao Y. (2019a). LncRNA MSC-AS1 promotes osteogenic differentiation and alleviates osteoporosis through sponging microRNA-140–5p to upregulate BMP2. Biochem. biophysical Res. Commun. 519 (4), 790–796. 10.1016/j.bbrc.2019.09.058 31551149

[B61] Zhang Y. Chen B. Li D. Zhou X. Chen Z. (2019b). LncRNA NEAT1/miR-29b-3p/BMP1 axis promotes osteogenic differentiation in human bone marrow-derived mesenchymal stem cells. Pathology-Research Pract. 215 (3), 525–531. 10.1016/j.prp.2018.12.034 30638953

[B62] Zhang Y. Xing Y. Jia L. Ji Y. Zhao B. Wen Y. (2018). An *in vitro* comparative study of multisource derived human mesenchymal stem cells for bone tissue engineering. Stem Cells Dev. 27 (23), 1634–1645. 10.1089/scd.2018.0119 30234437

[B63] Zhao Y. Ning J. Teng H. Deng Y. Sheldon M. Shi L. (2024). Long noncoding RNA Malat1 protects against osteoporosis and bone metastasis. Nat. Commun. 15 (1), 2384. 10.1038/s41467-024-46602-3 38493144 PMC10944492

[B64] Zhao Y. Richardson K. Yang R. Bousraou Z. Lee Y. K. Fasciano S. (2022a). Notch signaling and fluid shear stress in regulating osteogenic differentiation. Front. Bioeng. Biotechnol. 10, 1007430. 10.3389/fbioe.2022.1007430 36277376 PMC9581166

[B65] Zhao Y. Wang S. (2023). Detection of MicroRNA expression dynamics using LNA/DNA nanobiosensor. Methods Mol Biol. 2630, 75–87. 10.1007/978-1-0716-2982-6_6 36689177

[B66] Zhao Y. Yang R. Bousraou Z. Richardson K. Wang S. (2022b). Probing notch1-Dll4 signaling in regulating osteogenic differentiation of human mesenchymal stem cells using single cell nanobiosensor. Sci. Rep. 12, 10315. 10.1038/s41598-022-14437-x 35725756 PMC9209437

[B67] Zhou Z. Hossain M. S. Liu D. (2021). Involvement of the long noncoding RNA H19 in osteogenic differentiation and bone regeneration. Stem Cell Res. and Ther. 12 (1), 74–79. 10.1186/s13287-021-02149-4 33478579 PMC7819155

