# Supplementary material for: Involvement of long non-coding RNA (lncRNA) MALAT1 in shear stress regulated adipocyte differentiation
Source: Front Bioeng Biotechnol. 2025 May 6;13:1570518. doi: 10.3389/fbioe.2025.1570518 (PMC12089105; doi:10.3389/fbioe.2025.1570518)
Supplement: Supplementary file 1 [file DataSheet1.docx]

**Involvement of Long non-coding RNA (lncRNA) MALAT1 in Shear Stress regulated Adipocyte Differentiation**

Justin Caron ^1^, Marjan Ghanbariabdolmaleki ^1^, Madison Marino ^2^, Chong Qiu ^1^, Bo Wang ^3^, Michael Mak ^4^, Shue Wang ^1, *^

^1^Department of Chemistry, Chemical and Biomedical Engineering, University of New Haven, West Haven, CT, 06516, USA.

^2^Department of Forensic Science, University of New Haven, West Haven, CT, 06516, USA.

^3^Joint Department of Biomedical Engineering, Marquette University and the Medical College of Wisconsin, Milwaukee, WI 53226, USA

^4^Department of Pharmacological Sciences, Renaissance School of Medicine, Stony Brook University, Stony Brook, NY, USA.


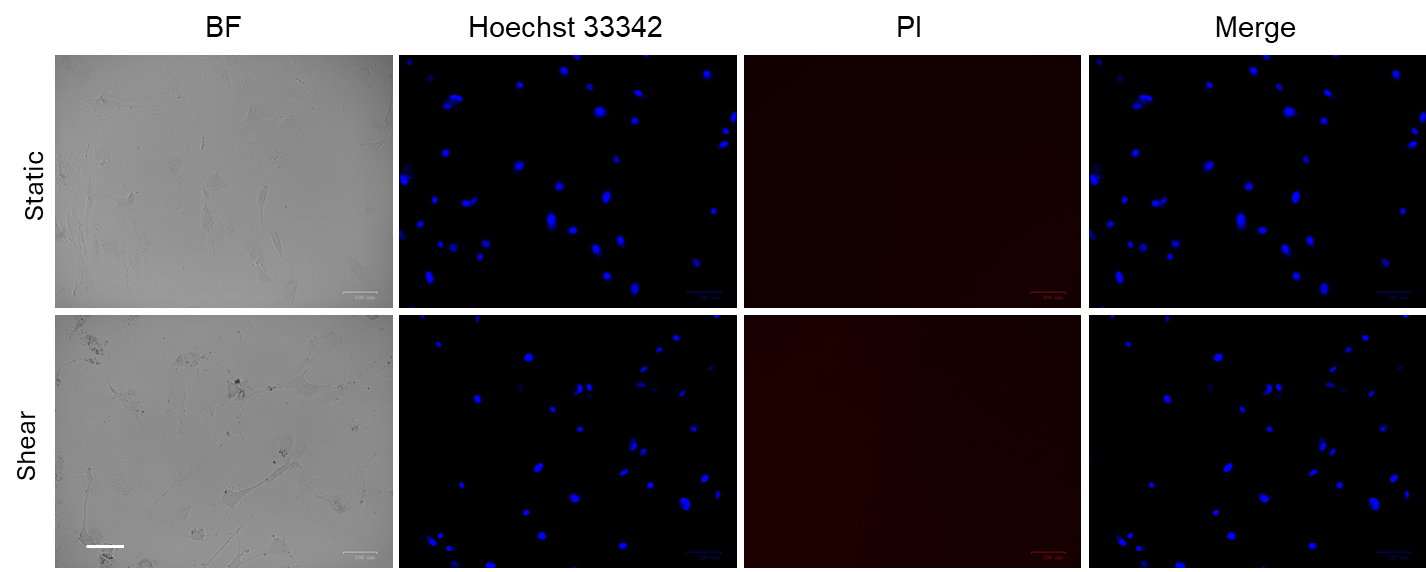


**Figure S1.** Representative images of MSCs under static and shear conditions after 5 days of culture. BF: Bright field, Hoechst 33342: Nucleus staining. Propidium Iodide (PI): stain dead cells. (n=3) Scale bar: 100 µm.


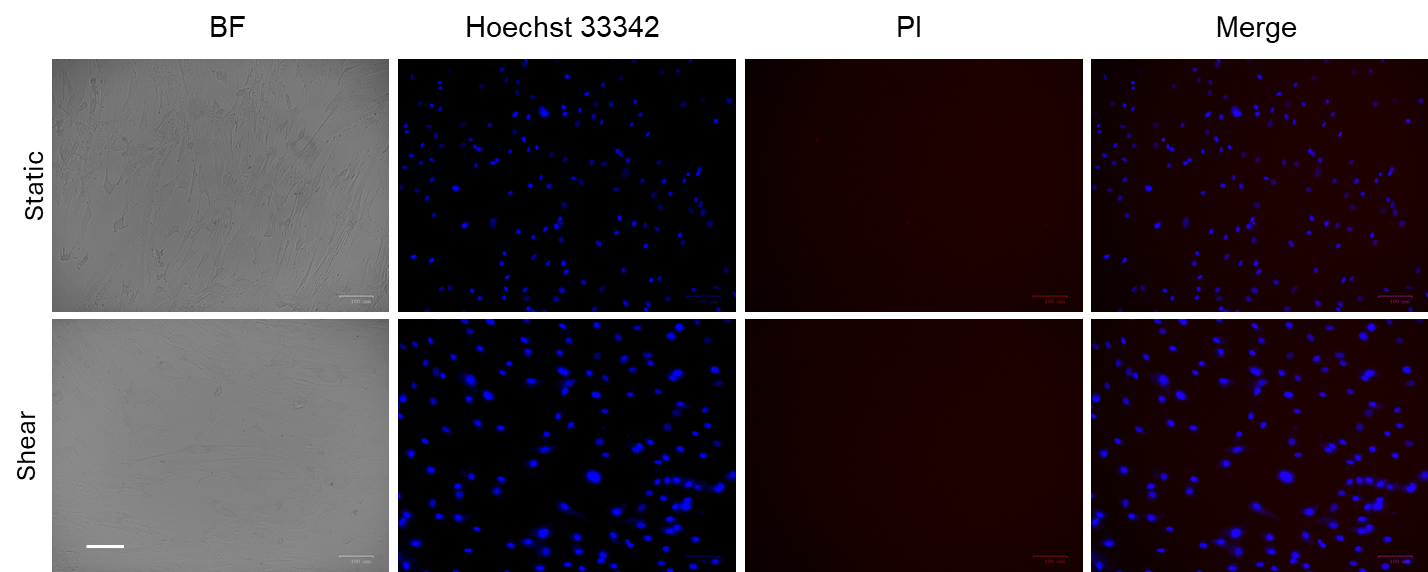


**Figure S2.** Representative images of MSCs under static and shear conditions after 10 days of culture. BF: Bright field, Hoechst 33342: Nucleus staining. Propidium Iodide (PI): stain dead cells. (n=3) Scale bar: 100 µm.


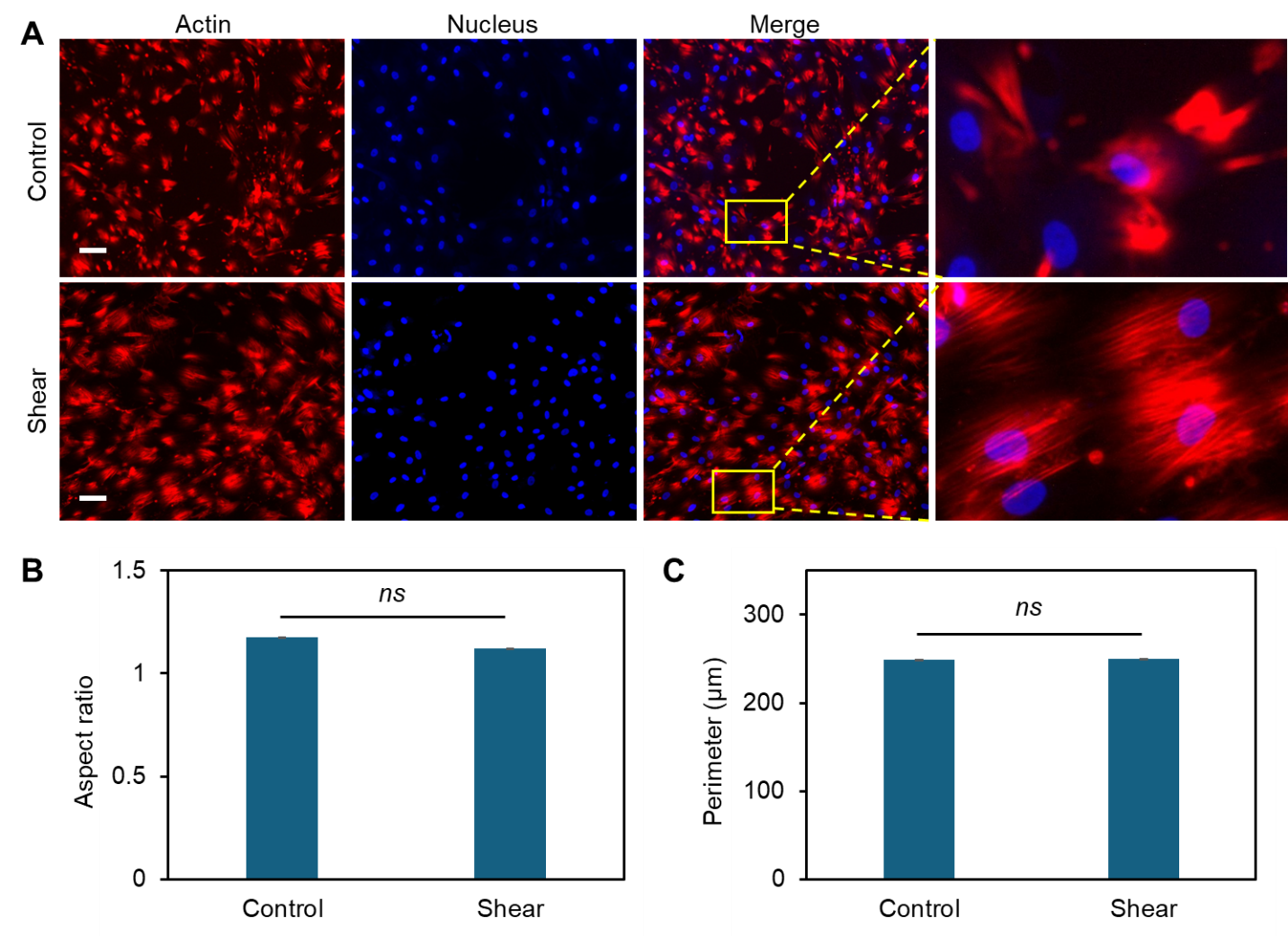


**Figure S3. Effects of shear stress on MSCs morphological changes and actin organization**. **(A)** Representative bright field and fluorescence images of MSCs under static condition (control) and exposed to shear stress (shear). MSCs were exposed to orbital shear continuously for 5 days. Samples were stained with F-actin (red; by phalloidin), and nuclei (blue; by Hoechst 33342), respectively. Scale bar: 100 μm. Comparison of aspect ratio **(B)** and perimeters **(C)** of MSCs after 10 days of exposure to low fluid shear. Data represents over 200 cells in each group and are expressed as mean± s.e.m. (n = 5, ***, p < 0.001, **, p < 0.01, *, p < 0.05).


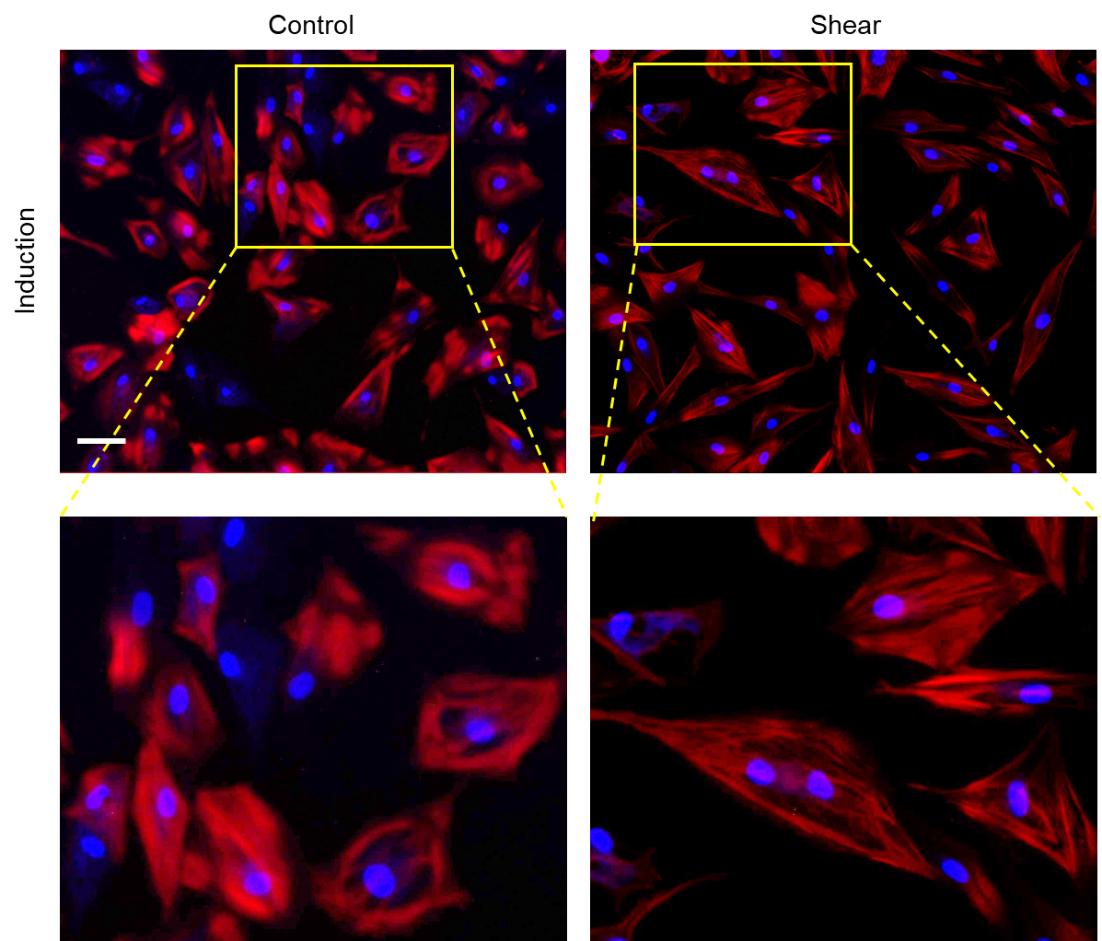


**Figure S4.** **Effects of shear stress on actin organization during adipocyte differentiation**. Red: F-actin. Blue: Nucleus. During adipocyte differentiation, actin cytoskeleton under drastic remodeling from aligned stress fibers to cortical actin structures. For cells exposed to shear stress, actin cytoskeleton remodeling was mediated and stress fibers maintained elongated inside cells.


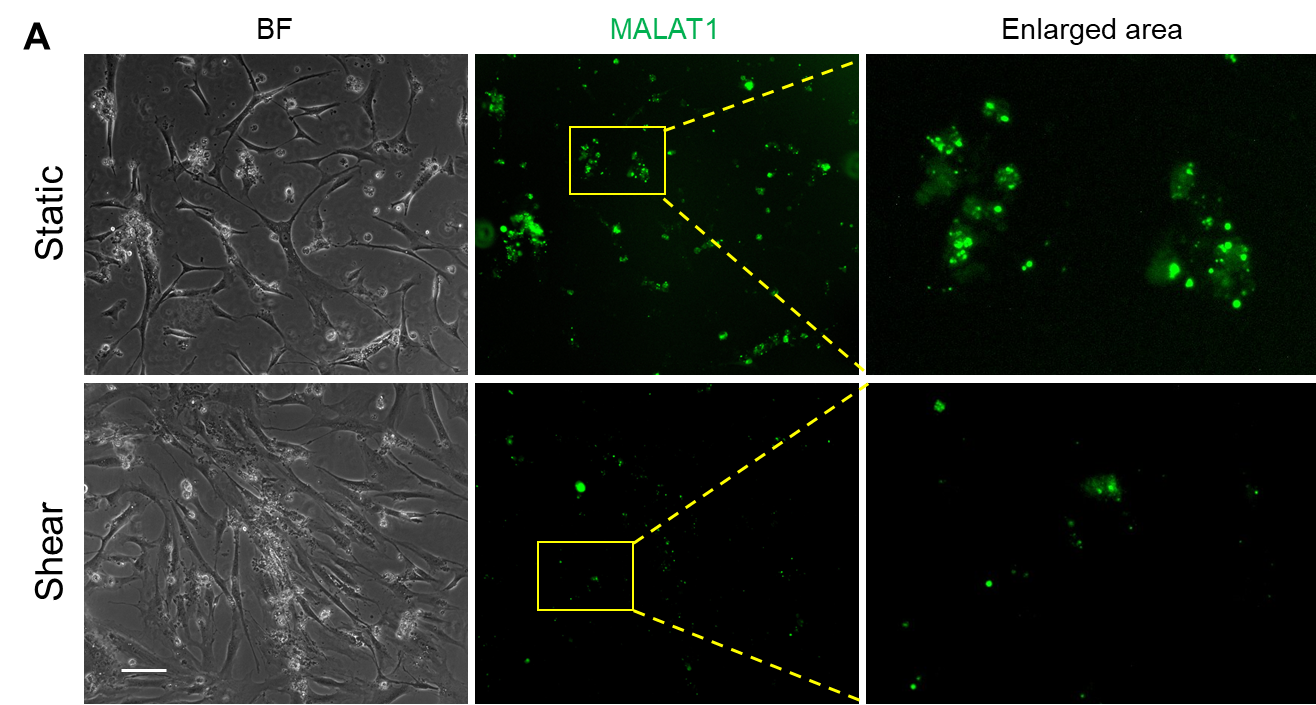


**Figure S5. Comparison of MALAT1 expression of hMSCs in basal medium (no induction).** **(A)** Representative images of hMSCs under static and shear conditions. Green fluorescence signal is MALAT1 expression.


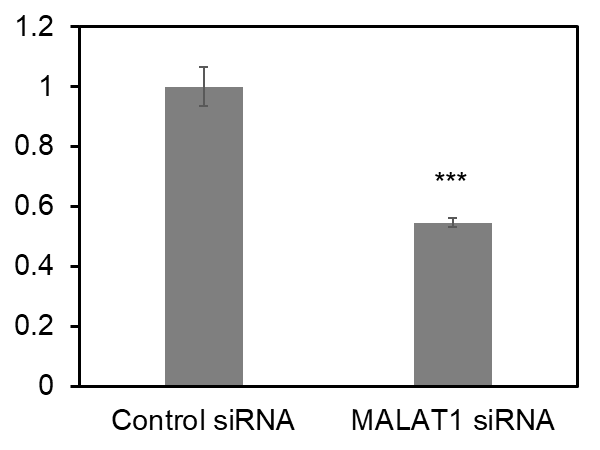


**Figure S6. MALAT1 siRNA knockdown efficiency.**  RT-PCR was performed to evaluate the silencing efficiency. The relative expression levels of MALAT1 were determined by the equation 2^−ΔΔCt^. Data are expressed as mean ± s.e.m. (n = 3). A two-tailed t-test was used to analyze differences between control siRNA and MALAT1 siRNA. ***, p < 0.005.

**Table S1. MALAT1 LNA probe sequences**

| Name | | Sequence (5’-3’) | Fluorophore |
| --- | --- | --- | --- |
| MALAT1 | Donor | +T+C+G+C+A TACGT GTGTC TGCTG AGTGT +T+C+C+T+G | /56-FAM |
|  | Quencher | +G+A+C+A+C ACGTA TGCGA | /3-Iowa BlackFQ |
